# Experimental Study on the Bearing Capacity of Fiber-Reinforced Geopolymer Pipes

**DOI:** 10.3390/ma19194141

**Published:** 2026-09-28

**Authors:** Shukang Ying, Lin Hu, Fang Xu, Peng Zhang, Wendong Fan, Wei Luo, Shichong Yang

**Affiliations:** 1 Department of Underground Space, School of Engineering, China University of Geosciences (Wuhan), Wuhan 430074, China; 2 Hubei Wujing Freeway Development Co., Ltd., Wuhan 430010, China

**Keywords:** geopolymer pipes, three-edge bearing load, PVA fibers, GFRP reinforcement

## Abstract

To investigate the mechanical behavior and failure mechanisms of fiber-reinforced geopolymer drainage pipes under three-edge bearing loads, three-edge bearing tests were conducted on unreinforced engineered geopolymer composite (EGC) and polyvinyl alcohol fiber-reinforced engineered geopolymer composite (PVA-EGC) pipes, as well as glass fiber-reinforced polymer (GFRP)-reinforced EGC and PVA-EGC pipes. The effects of different matrix materials and reinforcement types on the bearing capacity, crack development, and strain field evolution were systematically analyzed based on the load–displacement response, crack evolution, and distributed fiber optic monitoring. The results show that the unreinforced pipes exhibited brittle failure with four-lobe cracking. The bridging effect of PVA fibers delayed crack propagation and improved the post-cracking bearing capacity and structural integrity. The ultimate bearing capacity increased from 2489.38 N for the EGC pipe to 3088.98 N for the PVA-EGC pipe, representing an increase of 24.1%. For the reinforced pipes, the cracking load increased from 7215.18 N for the GFRP-reinforced EGC pipe to 15,339.56 N for the GFRP-reinforced PVA-EGC pipe, corresponding to an increase of 112.6%. The ultimate bearing capacity increased from 18,123.94 N to 24,855.69 N, representing an increase of 37.1%. The GFRP-reinforced PVA-EGC pipe exhibited a more gradual decrease in post-peak bearing capacity and improved deformation resistance and structural ductility. Crack analysis showed that PVA fibers effectively restrained crack localization. The cracking mode changed from through-cracks to multiple fine and uniformly distributed cracks, significantly improving the crack control capacity of the pipe. In addition, the combined action of PVA fibers and GFRP reinforcement reduced strain concentration and promoted more uniform stress transfer. This delayed crack propagation and the development of the GFRP reinforcement toward its ultimate stress state, thereby improving the overall bearing performance of the pipe.

## 1. Introduction

Underground pipeline networks are important infrastructure for urban operation. Their safety and durability have attracted increasing attention. Traditional reinforced concrete pipes have advantages such as low cost and mature construction methods. However, concrete deterioration and reinforcement corrosion can occur under sulfate and chloride attack, freeze–thaw cycles, and wastewater corrosion. These problems can reduce the bearing capacity of the pipes. In addition, concrete has limited crack resistance and impermeability. Crack propagation can cause leakage, soil and water loss, and uneven settlement. Under the combined effects of long-term loading and environmental conditions, the service performance of pipes may further deteriorate. Therefore, it is important to develop new pipe materials with high durability and good mechanical performance.

To address the above problems of traditional concrete materials, Engineering Geopolymer Composites (EGC) have received increasing attention as a new type of inorganic cementitious material. EGC uses industrial solid wastes such as fly ash and slag as precursors. Under alkali activation, these materials form a dense three-dimensional network structure through depolymerization and polycondensation reactions [1,2]. Studies have shown that an appropriate alkali activation system can promote geopolymerization and improve the compactness of the internal structure. This can significantly improve the mechanical and durability properties of the material [3]. In addition, active components such as slag can provide a calcium source. This accelerates the reaction and improves the early strength. Huang [4], Rajamane [5], and Zhang et al. [6] reported that geopolymers have good stability in acidic, sulfate, and chloride environments. Their corrosion resistance is significantly better than that of ordinary Portland cement concrete. Mao Jieming [7] found through orthogonal tests that the compressive strength of geopolymer concrete can exceed 70 MPa under suitable curing conditions and an appropriate mix ratio. This is much higher than that of traditional reinforced concrete. In addition, replacing cement with industrial solid wastes such as fly ash and slag can improve material performance and significantly reduce carbon emissions. This provides good environmental benefits [8]. However, ordinary geopolymer materials still show obvious brittleness under tension. Their crack resistance is also limited. These limitations restrict their application in flexural and other complex structures.

To overcome these limitations, fiber reinforcement has been widely used to modify geopolymer materials. One approach is to add high-performance fibers such as polyvinyl alcohol (PVA) fibers. This can produce high-ductility geopolymer composites with strain-hardening behavior. Their crack resistance and deformation capacity can therefore be significantly improved. Li Mingyang [9] reported that fiber addition can effectively reduce material brittleness. The ductility of the material also increases significantly with increasing fiber content. Yu [10] found that the ultimate tensile strain of PVA-EGC can reach 3.6–4.5%, while the crack width can be controlled within a small range. Cui [11] further reported that these materials develop multiple fine cracks under flexural loading. They also show good deformation and energy dissipation capacities. Lepech [12] and Guo [13] reported that EGC materials have good durability and crack control during long-term service. These properties indicate good potential for engineering applications.

Another effective approach is to combine Fiber Reinforced Polymer (FRP) rebar with fiber-reinforced geopolymer materials. Their combined action can improve material ductility and crack control. This can achieve coordinated improvement in durability and mechanical performance at the structural level and allow the material to withstand more complex loading conditions [14]. FRP rebar has the advantages of low weight, high strength, corrosion resistance, and good environmental adaptability. Among them, Glass Fiber Reinforced Polymer (GFRP) rebar is widely used in underground and water-related engineering because of its relatively good economic performance [15]. However, when FRP rebar is used alone as the reinforcement, its low elastic modulus and lack of yielding can result in insufficient structural ductility. Large deformation and rapid crack development may occur during service [16,17,18,19,20,21]. Therefore, FRP rebar alone is not sufficient to meet the combined requirements for deformation capacity and crack control. It is necessary to use FRP rebar together with fiber-reinforced materials.

Although considerable achievements have been obtained for geopolymer materials and fiber-reinforced composites, most existing studies focus on beams, slabs and columns. Limited research is available concerning the mechanical behavior, crack evolution and failure mechanisms of fiber-reinforced geopolymer drainage pipes under three-edge bearing loads. Accordingly, this work investigates fiber-reinforced geopolymer drainage pipes. Using EGC and PVA-EGC as pipe matrices and adopting GFRP reinforcement, unreinforced and reinforced pipe specimens were fabricated, and three-edge bearing tests were carried out. Based on load–displacement responses and crack development patterns, the effects of PVA fibers and GFRP reinforcement on pipe bearing capacity, crack control, strain-field evolution and failure modes were systematically analyzed. The load-bearing mechanism of pipes under the combined effect of fibers and GFRP reinforcement was also explored. The findings can provide a theoretical basis and technical references for the structural design and engineering application of fiber-reinforced geopolymer drainage pipes, as well as for the development of green high-performance underground drainage systems. It should be noted that the conclusions are derived from test data of GFRP-bar/PVA-EGC pipes with the above-mentioned specific mix proportions. They offer direct references only for the structural design of pipes within this material system. Further verification via corresponding material-scale tests is required if these conclusions are to be extended to other mix proportions or material systems.

## 2. Experimental Program

### 2.1. Pipe Design and Fabrication

This study focuses on the mechanical performance of fiber-reinforced geopolymer drainage pipes under three-edge bearing loads, mainly examining the effects of matrix type and reinforcement form on the bearing capacity and failure characteristics of the pipes. The EGC matrix used in this study consisted of 42.5-grade ordinary Portland cement (Xinfeng Industrial Park, Yangcun Township, Hejin, Yuncheng, China), S95-grade ground granulated blast-furnace slag (Xinfeng Industrial Park, Yangcun Township, Hejin, Yuncheng, China), Class I fly ash (Xinfeng Industrial Park, Yangcun Township, Hejin, Yuncheng, China), quartz sand (Tianjin Yandong Mining, Tianjin, China), metakaolin (Xinfeng Industrial Park, Yangcun Township, Hejin, Yuncheng, China), silica fume (Xinfeng Industrial Park, Yangcun Township, Hejin, Yuncheng, China), water, and an alkaline activator. PVA-EGC was prepared by adding high-modulus PVA fibers (Anhui Wanwei Updated High-Tech Material Industry Co., Ltd., Chaohu, China) to the same EGC matrix. The S95-grade slag had a density of 2.88 g/cm^3^ and a specific surface area of 440 m^2^/kg. The Class I fly ash had a density of 2.14 g/cm^3^ and a specific surface area of 2350 m^2^/kg. The quartz sand had a particle size of 20–40 mesh and a hardness of 7. The alkaline activator consisted of sodium silicate (Bengbu Jingcheng Chemical Co., Ltd., Bengbu, China) and sodium hydroxide (Shandong Binhua Dongrui Chemical Co., Ltd., Binzhou, China). The sodium silicate had a modulus of 3.23, and the sodium hydroxide had a purity of 95%. The PVA fibers had a length of 12 mm and a diameter of 32 μm, with a tensile strength of 1600 MPa, an elastic modulus of 35 GPa, and an elongation at break of 6.5 ± 1%. The material proportions of PVA-EGC were calculated based on the mass of the alkaline activator. The mass ratios of fly ash, metakaolin, silica fume, slag, PVA fibers, and water to the alkaline activator were 34%, 25%, 5%, 22%, 2%, and 12%, respectively. EGC and PVA-EGC pipes with different diameters, wall thicknesses, and GFRP reinforcement (Shandong Daitian Engineering Materials Co., Ltd., Taian, China) conditions were designed. Four groups of pipe specimens were prepared for the loading tests. One pipe was cast for each group, resulting in a total of four pipes. After casting, the pipes were left under natural curing conditions for 24 h before demolding. All specimens were then cured indoors for 28 d. During the curing period, the surfaces of the pipes were regularly inspected for cracks, and water was periodically sprayed to maintain suitable curing conditions. The pipe parameters were designed with reference to GB 11836-2023, Concrete and Reinforced Concrete Drainage Pipes [22], and Design Manual for Structural Dimensions and Reinforcement of Reinforced Concrete Drainage Pipes [23]. The detailed parameters of the two unreinforced pipe groups are listed in Table 1. The detailed parameters of the two reinforced pipe groups are listed in Table 2. The shear and tensile strengths of the GFRP reinforcement were 124 MPa and 786 MPa, respectively. Its elastic modulus and ultimate tensile strain were 42 GPa and 1.68%, respectively. The material properties of PVA-EGC and EGC are listed in Table 3 and Table 4, respectively.

### 2.2. Pipe Loading Test

The loading test was conducted using the indoor three-edge bearing test method, with reference to the circumferential bearing capacity test method for rigid pipes specified in GB/T 16752-2017, Test Methods for Concrete and Reinforced Concrete Sewer Pipes [24]. The loading setup is shown in Figure 1.

Displacement control was used during the test on a universal testing machine (Instron, Norwood, MA, USA; software Bluehill Universal v4.55). The ultimate bearing capacity of each specimen was first estimated based on the preliminary test results. The loading procedure was then determined. For the unreinforced pipes, the loading rate was set to 1.5 mm/min and maintained until failure. For the reinforced pipes, the loading process was divided into three stages to better observe crack evolution and failure characteristics. The loading rates were 1.5 mm/min, 3.0 mm/min, and 4.5 mm/min, respectively.

The test was terminated when the displacement reached 10% of the nominal pipe diameter. The data acquisition frequency was set to 1 Hz throughout the test. This ensured continuous and complete collection of the load and displacement responses.

To accurately monitor the mechanical response, deformation and failure process of the pipes under three-edge bearing loads, a strain measurement method combining distributed fiber optics and foil strain gauges was used. A LUNA distributed fiber optic sensing system (Luna Innovations Inc., Roanoke, VA, USA; ODiSI 6000 series; ODiSI Application Software v2.4.3) was used to obtain the hoop strain distribution on the inner and outer surfaces of the pipe wall in real time. Foil strain gauges (KYOWA Electronic Instruments, Tokyo, Japan) were also installed at key locations, including the pipe bottom, pipe top, and both pipe haunches, to monitor the local hoop strain simultaneously. The optical fiber was bonded using UV-curable adhesive (LOCTITE, Henkel AG & Co. KGaA, Düsseldorf, Germany), with free sections of at least 200 mm reserved at both ends. The strain gauges were installed in the same direction as the fiber optic measurement path. This enabled coordinated monitoring of the overall and local mechanical responses of the pipe. The arrangement is shown in Figure 2.

## 3. Analysis of Test Results

### 3.1. Results Analysis of Unreinforced Pipes

#### 3.1.1. Failure Mode

A1 and A2 were used as unreinforced comparison specimens. The test results showed that both groups exhibited typical brittle failure. During loading, no obvious macroscopic deformation was observed. When the load reached the ultimate value, the pipes suddenly cracked at the crown, invert, and both haunches. The pipes finally split into four parts and experienced crushing failure. The failure modes are shown in Figure 3a. In contrast, the bridging effect of PVA fibers in A2 effectively restrained rapid crack propagation. Although the pipe also developed four-lobe cracking at failure, no overall collapse occurred. The pipe retained a certain degree of structural integrity. Its failure mode is shown in Figure 3b.

#### 3.1.2. Load–Displacement Analysis

The load–displacement curves of the unreinforced pipes in Groups A1 and A2 are shown in Figure 4. Overall, both groups experienced three stages: elastic response, nonlinear development, and failure. However, their load–displacement responses were significantly different. For Group A1 (EGC pipe), the load increased continuously with displacement during the initial loading stage. When the load reached 2489.38 N, the specimen failed. The load then decreased rapidly, showing typical brittle failure. The specimen had almost no post-cracking bearing capacity. In contrast, the load increased more slowly for Group A2 (PVA-EGC pipe) during the initial loading stage. As the displacement increased, the bridging effect of PVA fibers gradually became significant. The specimen then entered a clear post-cracking strengthening stage, and the bearing capacity continued to increase. The ultimate load reached 3088.98 N, which was about 24.1% higher than that of A1. After reaching the peak load, the load–displacement curve of A2 did not decrease sharply. Instead, it showed a fluctuating and gradual decrease. This indicates that fiber pullout and fiber rupture occurred during crack propagation. The bridging effect continuously dissipated external energy and allowed the specimen to maintain a high post-cracking bearing capacity. As the bridging effect gradually weakened, the bearing capacity slowly decreased. However, the specimen still retained some residual bearing capacity. These results show that PVA fibers significantly improved the post-cracking bearing capacity, deformation capacity, and toughness of the EGC pipe.

#### 3.1.3. Hoop Strain Analysis

The hoop strain at eight measurement points on the inner and outer surfaces of each pipe was measured using distributed fiber optics and strain gauges. The pipe bottom was defined as 0°. Measurement points were arranged at 0°, 90°, 180°, and 270° on both the inner and outer surfaces. The hoop strains measured by the distributed fiber optics and strain gauges at the failure load of the Group A pipes were extracted for comparison, as shown in Figure 5.

The strain measurements from the strain gauges and distributed fiber optics show that the strains at the critical points were consistent with the brittle failure mode of the pipes at the failure load. For the A2 pipe, the hoop strain at the inner surface at 0° reached 2338 με. This is consistent with the obvious cracking at the 0° position observed in the A2 pipe during the three-edge bearing test. The tensile contribution of the PVA fibers helped maintain the structural integrity of the pipe, and the pipe did not collapse after failure.

#### 3.1.4. Crack Pattern and Development

During the initial loading stage, the specimens were in the elastic stage. The displacement increased steadily with the load, and no obvious cracks appeared on the surface. When the load reached the cracking load, cracks first appeared on the outer surfaces of the left and right haunches. Cracks then appeared on the inner surfaces of the crown and invert. These locations were consistent with the regions of peak hoop tensile strain. Therefore, these areas were closely monitored during the test. As the load continued to increase, the cracks in both groups of unreinforced pipes developed rapidly. Both pipes finally exhibited brittle failure. Through-cracks formed at the haunches, as shown in Figure 6. Compared with A1, A2 (PVA-EGC) showed slower crack propagation due to the bridging effect of PVA fibers. The pipe also retained a certain post-cracking bearing capacity after reaching the peak load. The pipe did not split into four parts at the final stage. However, as the bridging effect gradually weakened, the crack width continued to increase, and brittle failure eventually occurred. These results indicate that PVA fibers can effectively restrain crack propagation and improve the post-cracking bearing capacity and structural toughness. However, they did not change the dominant brittle failure mode of the unreinforced pipes. The safety reserve was therefore limited.

### 3.2. Results Analysis of Reinforced Pipes

#### 3.2.1. Failure Mode

There were clear differences in the failure modes between the B1 and B2 pipes. During loading, the B1 pipe developed a large number of cracks. The cracks also propagated rapidly. After reaching the ultimate load, the GFRP reinforcement fractured, and the bearing capacity of the pipe was rapidly lost. The pipe therefore showed obvious brittle failure. The experimental failure mode of B1 is shown in Figure 7a. After reaching the ultimate load, cracks in the tensile region rapidly developed and became localized. The pipe then failed suddenly with the fracture of the GFRP reinforcement. This was mainly because the EGC matrix without PVA fibers had limited crack resistance. Once cracks formed, they could rapidly propagate and become localized. In addition, GFRP reinforcement has a low elastic modulus and linear elastic behavior. It has no yielding stage for energy dissipation. Its smooth surface also provides relatively weak mechanical interlocking with the EGC matrix. The bond performance at the interface was therefore limited. Slip of the reinforcement or interface debonding could occur during loading. As a result, cracks were not effectively controlled, and the specimen eventually failed suddenly.

In contrast, cracks in the B2 pipe first appeared near the loading zone at the crown. They then gradually extended toward both sides. As the load increased, more fine cracks developed on the pipe surface. The cracks were relatively evenly distributed, and no obvious crack localization occurred. The experimental failure mode of B2 is shown in Figure 7b. Near the ultimate load, the main crack at the crown loading point rapidly widened and extended along the outer circumference of the pipe. The PVA-EGC matrix in the compression zone gradually crushed and spalled. A clear opening angle then formed, and the pipe reached its ultimate bearing capacity and failed. The final failure mode shows that the pipe retained good overall integrity. Only local crushing occurred in the loading zone. A large number of fine cracks were distributed in the tensile region. No single macroscopic crack penetrated the entire cross-section. This was mainly due to the bridging effect of PVA fibers. The fibers effectively restrained rapid crack propagation. They converted strain concentration into the coordinated development of multiple cracks. The cracks therefore became finer and more uniformly distributed. This improved the damage tolerance and deformation capacity of the pipe. At the same time, the GFRP reinforcement continued to carry tensile stress. It worked together with the PVA-EGC matrix. The crack development was therefore more gradual during failure, and the bearing capacity decreased progressively. The pipe showed relatively clear ductile failure characteristics rather than sudden brittle failure. The final failure modes of the two groups are shown in Figure 7.

#### 3.2.2. Load–Displacement Analysis

The load–displacement curves of the reinforced pipes in Groups B1 and B2 are shown in Figure 8. Both groups experienced three stages: elastic response, cracked stage, and failure. However, their mechanical responses and failure characteristics were significantly different because of the different matrix materials.

Group B1 used a GFRP-reinforced EGC structure. During the initial loading stage, the pipe was in the elastic stage. The load–displacement curve increased approximately linearly. However, slight fluctuations occurred due to the initiation and propagation of microcracks in the EGC matrix. The overall slope was relatively small. When the crack width reached 0.2 mm, the cracking load was 7215.18 N. The pipe then entered the cracked stage. As the load increased, the number of cracks increased, and the existing cracks gradually propagated. The pipe stiffness gradually decreased, but the bearing capacity continued to increase. When the load reached 18,123.94 N, the pipe reached its ultimate bearing capacity. The GFRP reinforcement in the tensile region fractured, and the bearing capacity was rapidly lost. The load–displacement curve then dropped sharply, showing obvious brittle failure.

In contrast, Group B2 used a GFRP-reinforced PVA-EGC structure. The addition of PVA fibers significantly improved the crack resistance of the matrix. The cracking load increased to 15,339.56 N, which was 112.6% higher than that of B1. The load–displacement curve had a larger overall slope, indicating a significant increase in the initial stiffness of the pipe. After entering the cracked stage, the PVA-EGC matrix and GFRP reinforcement worked together. Due to the bridging effect of PVA fibers, cracks developed as fine and multiple cracks with a relatively uniform distribution. Crack propagation was effectively restrained. The load–displacement curve therefore changed more gradually, showing good deformation compatibility. When the load reached 24,855.69 N, the pipe reached its ultimate bearing capacity. This was 37.1% higher than that of B1. After the peak load, the bearing capacity of B2 decreased gradually. No sharp decrease occurred due to GFRP reinforcement fracture. Even when the vertical deformation reached 10% of the pipe diameter, the specimen still maintained good overall integrity and bearing capacity. It showed better deformation resistance and more obvious ductile failure characteristics than B1. This was mainly because the bridging effect of PVA fibers effectively restrained crack initiation and propagation. It transformed local strain concentration into the coordinated development of multiple cracks. Energy was also continuously dissipated through fiber pullout. This improved the toughness and crack control capacity of the matrix. At the same time, the distributed development of cracks made the stress in the GFRP reinforcement more uniform and reduced local stress concentration. This delayed the GFRP reinforcement from reaching its ultimate stress state. The combined action of the PVA-EGC matrix and GFRP reinforcement was therefore enhanced. As a result, the bearing capacity, deformation resistance, and structural ductility of the pipe were significantly improved.

#### 3.2.3. Hoop Strain Analysis

The hoop strain distributions of the Group B pipes at the failure load are shown in Figure 9. The compression and tension zones at the crown and invert of the pipe are 150–210° and 330–30°, respectively. The compression and tension zones on the left and right sides of the pipe are 30–120° and 240–330°, respectively. The pipe bottom was defined as 0°, and the angles were measured clockwise.

For the B1 pipe, the hoop strain measured by the inner optical fiber formed sharp peaks of approximately 4000–5000 με in the 150–180° sector near the pipe crown. The strain measured by the outer optical fiber remained around 3000 με throughout the circumference. A steep strain gradient was observed across the pipe section. This distribution is consistent with the failure process of B1. The EGC matrix without PVA fibers had limited crack resistance. Once a crack initiated, it rapidly propagated and became localized. The section deformation therefore concentrated at a single macroscopic crack. The outer optical fiber crossing the crack recorded a sharp increase in strain and formed a pronounced peak. The other regions remained at relatively low stress levels and contributed little to the overall load bearing. The GFRP reinforcement had a low elastic modulus and remained linearly elastic until fracture. It had no yielding stage for energy dissipation. In addition, its smooth surface resulted in weak mechanical interlocking with the EGC matrix. The interface bond performance was therefore limited. Reinforcement slip or interface debonding could occur during loading. The high strain peak in B1 was therefore not caused by overall plastic deformation of the pipe. Instead, it reflected localized strain concentration before brittle instability. After the ultimate load was reached, the GFRP reinforcement fractured, and the bearing capacity was rapidly lost.

For the B2 pipe, the hoop strain measured by the inner optical fiber remained at a relatively high level of 2000–3000 με around the entire circumference. The distribution was relatively uniform. The strain measured by the outer optical fiber remained around 3000–4000 με and changed gradually along the circumference. This feature agrees with the failure process of B2, which showed fine and uniformly distributed cracks without obvious localization. The bridging effect of PVA fibers restrained the rapid growth of a single crack and transformed localized strain concentration into the coordinated development of multiple cracks. The width of each crack was relatively small. The strain was therefore distributed over a wider circumferential range. The inner optical fiber consequently showed a gradual strain distribution around the circumference. The outer optical fiber also showed a wide region of relatively high strain rather than an isolated peak at a single location. This indicates that as the load increased, the pipe could gradually release local stress concentration through deformation and crack development over a relatively large region. The damage tolerance and deformation capacity of the pipe were therefore improved.

Overall, the hoop strain distributions of B1 and B2 showed significantly different failure characteristics. In B1, the strain was highly concentrated in a local region and showed sharp peaks. This reflected rapid crack propagation and localized section deformation. The GFRP reinforcement eventually fractured, and the bearing capacity was rapidly lost. The pipe therefore exhibited typical brittle failure. In contrast, B2 maintained relatively high and more uniform strain over a larger circumferential range. This indicates that the bridging effect of PVA fibers restrained crack localization and redistributed the strain through the coordinated development of multiple cracks. Local strain concentration was therefore reduced, and the damage tolerance and deformation capacity of the pipe were improved. Therefore, the addition of PVA fibers changed the strain transfer and damage evolution mechanisms of the pipe. The failure mode of B2 changed from brittle failure controlled by local strain concentration to ductile failure characterized by coordinated deformation over multiple regions.

#### 3.2.4. Crack Pattern and Development

During the three-edge bearing test, the B1 pipe showed early crack initiation, rapid crack propagation, and a tendency to form through-cracks. When the load increased to 7215.18 N, a crack first appeared on the inner surface of the pipe crown and rapidly widened to 0.2 mm, as shown in Figure 10a. Cracks then appeared on the inner surface of the invert and the outer surfaces of the left and right haunches. A multiple-crack pattern gradually formed in the tensile regions of the crown, invert, and haunches.

Because the EGC matrix without PVA fibers had limited crack resistance, the cracks propagated rapidly after initiation. The crack widths continued to increase, and the cracks gradually extended into the pipe wall and surrounding areas. The cracks also gradually connected with each other. As loading continued, new cracks formed in the tensile region, while existing cracks further propagated. The number and width of cracks increased simultaneously. Several through-cracks finally formed. These cracks were mainly concentrated on the inner surfaces of the crown and invert and the outer surfaces of the left and right haunches.

At the ultimate load, the crack widths further increased. Local spalling of the EGC matrix occurred at the crown and invert. The cracks penetrated the entire pipe wall and were eventually accompanied by fracture of the GFRP reinforcement. The specimen then lost overall stability and showed typical brittle failure. The crack pattern at this stage is shown in Figure 10b.

For the B2 pipe, the pipe showed good deformation resistance during the initial loading stage. As the load gradually increased, cracks first appeared in the tensile region on the inner surface of the invert and the outer surface of the right haunch. Unlike B1, the initial cracks did not rapidly propagate along the existing cracks. Instead, under the bridging effect of PVA fibers, the cracks gradually developed toward multiple tensile regions around the pipe. A fine, uniform, and stable multiple-crack pattern was formed. When the crack width reached 0.2 mm, the cracking load was 15,339.56 N. The crack pattern is shown in Figure 11a. Compared with B1, the cracking load of B2 increased by 112.6%. This indicates that PVA fibers significantly improved the crack resistance of the EGC matrix and effectively delayed crack initiation and propagation. PVA fibers also restrained crack localization. This allowed the tensile stress to be distributed more uniformly between the PVA-EGC matrix and the GFRP reinforcement. The development of the GFRP reinforcement toward its ultimate stress state was therefore delayed, and the crack control capacity of the pipe was improved.

As loading continued, the number of cracks increased continuously. However, crack propagation remained gradual, and no macroscopic crack penetrated the pipe wall. Meanwhile, the PVA-EGC matrix in the compression zone at the crown gradually developed layered and intersecting compressive deformation. The most severe damage occurred in this region. However, no obvious matrix spalling occurred. The compressive failure mode was consistent with the results of the PVA-EGC cube compressive tests. When the specimen reached the ultimate load, the vertical deformation reached 7.5% of the pipe diameter. The number of cracks increased significantly, and the maximum crack width reached 3.64 mm. However, the cracks did not penetrate the entire pipe wall. The crack failure mode at the ultimate load is shown in Figure 11b.

Compared with B1, which developed multiple through-cracks with obvious matrix spalling and GFRP reinforcement fracture, B2 showed a more uniform crack development pattern. The cracks did not penetrate the entire pipe wall, and the specimen maintained good overall integrity. These results indicate that PVA fibers effectively delayed crack penetration and local failure. The failure mode therefore changed from the rapid brittle failure of B1 to a ductile failure mode characterized by the coordinated development of multiple cracks.

## 4. Discussion and Conclusions

In this study, indoor three-edge bearing tests were conducted on unreinforced EGC and PVA-EGC pipes and GFRP-reinforced EGC and PVA-EGC pipes. The effects of pipe size, matrix type, and reinforcement on the flexural performance and bearing capacity of the pipes were investigated. The failure modes, load–displacement curves, and crack patterns were analyzed to evaluate the overall performance of the pipes. The main conclusions are as follows:

1. PVA fibers and GFRP reinforcement significantly improved the failure behavior of EGC pipes. The unreinforced pipes exhibited four-lobe brittle failure, whereas their combined action promoted more uniform stress transfer, reduced strain concentration, and shifted the failure mode toward progressive damage characterized by local crushing and multiple fine cracks.
2. Load–displacement analysis showed that PVA fibers and GFRP reinforcement significantly improved the bearing and deformation capacities of the pipes. For the unreinforced pipes, the addition of PVA fibers increased the ultimate bearing capacity by 24.1% and resulted in a more gradual post-peak load reduction. For the reinforced pipes, the GFRP-reinforced PVA-EGC pipe exhibited increases of 112.6% in cracking load and 37.1% in ultimate bearing capacity compared with the GFRP-reinforced EGC pipe. It also maintained stable load-carrying capacity when the vertical deformation reached 10% of the pipe diameter, indicating improved post-cracking deformation capacity and damage tolerance.
3. Crack evolution analysis showed that PVA fibers delayed crack propagation and improved crack control. In the unreinforced pipes, cracks initiated at the haunches and subsequently developed toward the crown and invert, eventually forming through-cracks. The GFRP-reinforced EGC pipe exhibited relatively early crack initiation and rapid crack propagation, accompanied by GFRP fracture. In contrast, the GFRP-reinforced PVA-EGC pipe showed a 112.6% increase in cracking load and developed multiple fine and more uniformly distributed cracks, which delayed crack penetration and local damage.
4. Future studies should further investigate the pipe–soil interaction and long-term durability of fiber-reinforced geopolymer drainage pipes under realistic buried and environmental conditions through large-scale tests and numerical simulations.

## Figures and Tables

**Figure 1:**
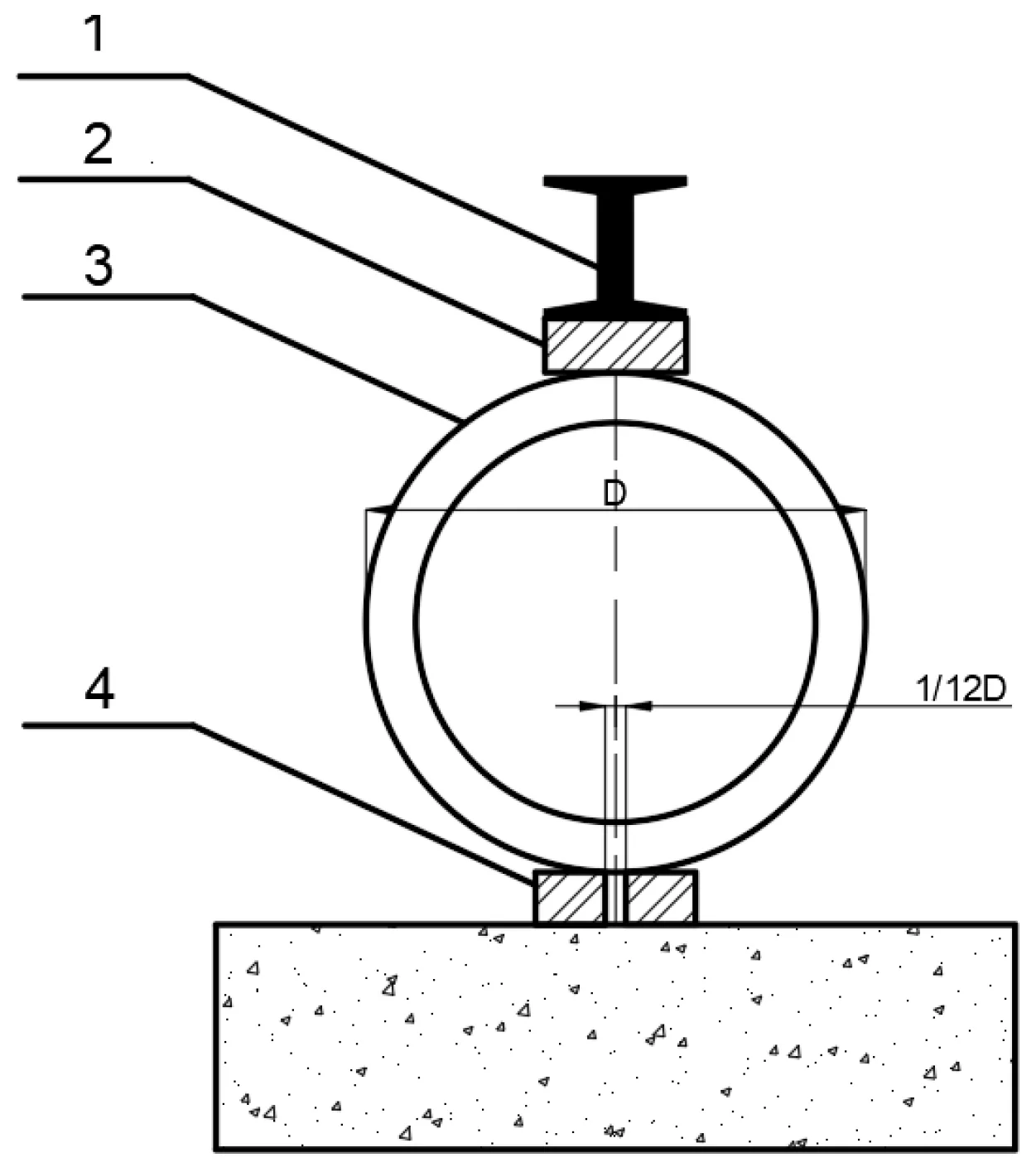
Schematic Diagram of the Loading Setup: 1—Steel upper supporting beam; 2, 4—Hardwood cushion strips; the spacing between two hardwood strips shall be 1/12 of the outer diameter of the pipe specimen on the plane perpendicular to the pressure-testing direction, and shall not be less than 25 mm. For pipe outer diameters of 250 mm and 400 mm in this test, the corresponding spacings are 25 mm and 35 mm, respectively; 3—Test specimen.

**Figure 2:**
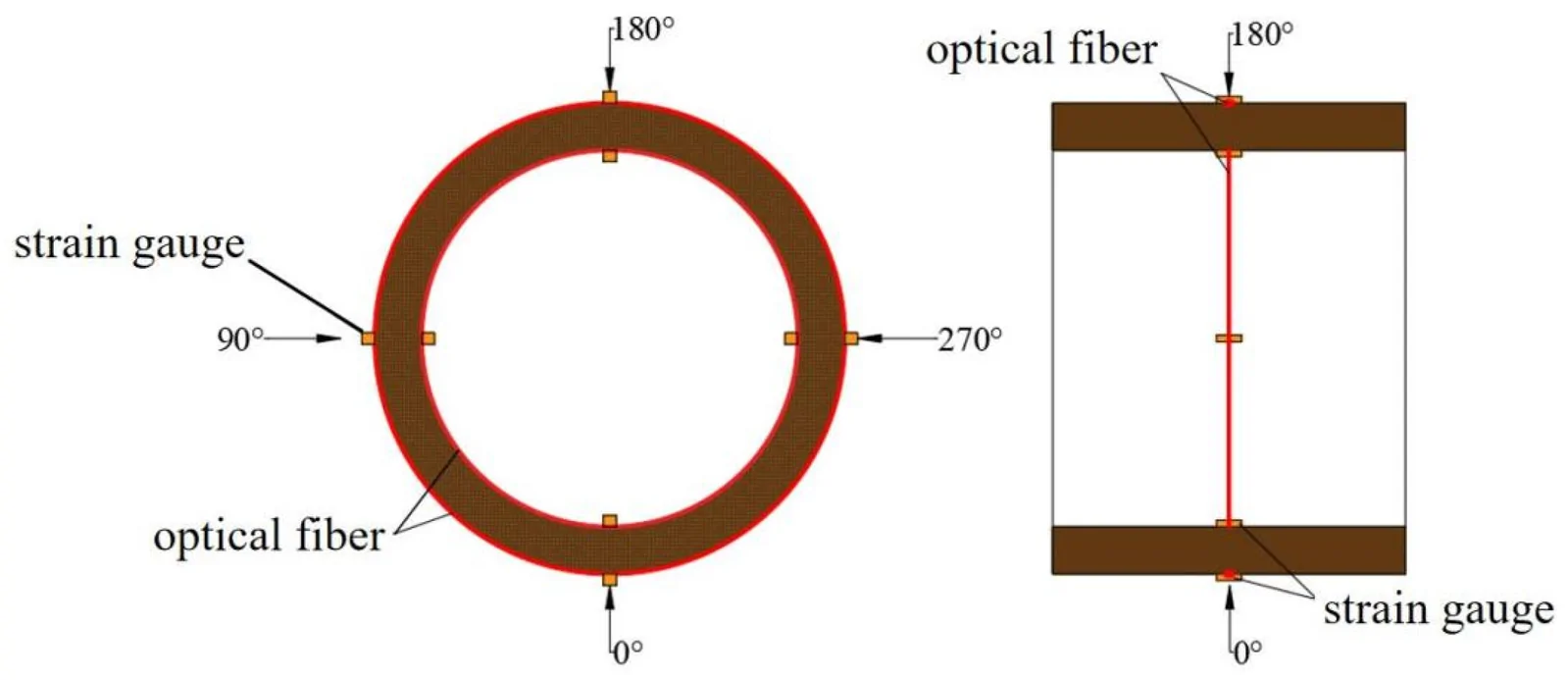
Schematic Diagram of Strain Gauge and Distributed Fiber Optic Measurement Point Arrangement.

**Figure 3:**
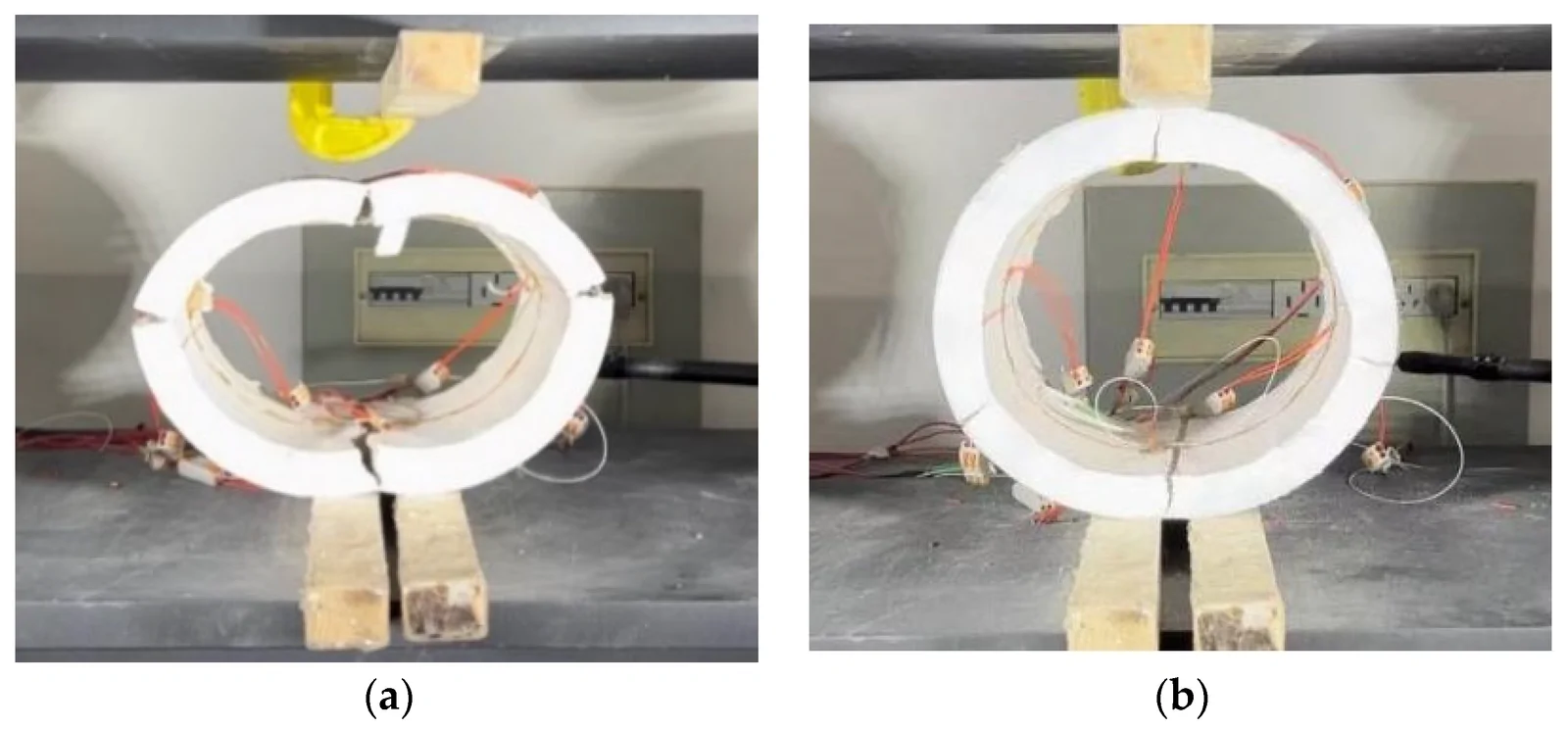
Experimental failure modes of Group A pipes: (**a**) Experimental failure of A1; (**b**) experimental failure of A2.

**Figure 4:**
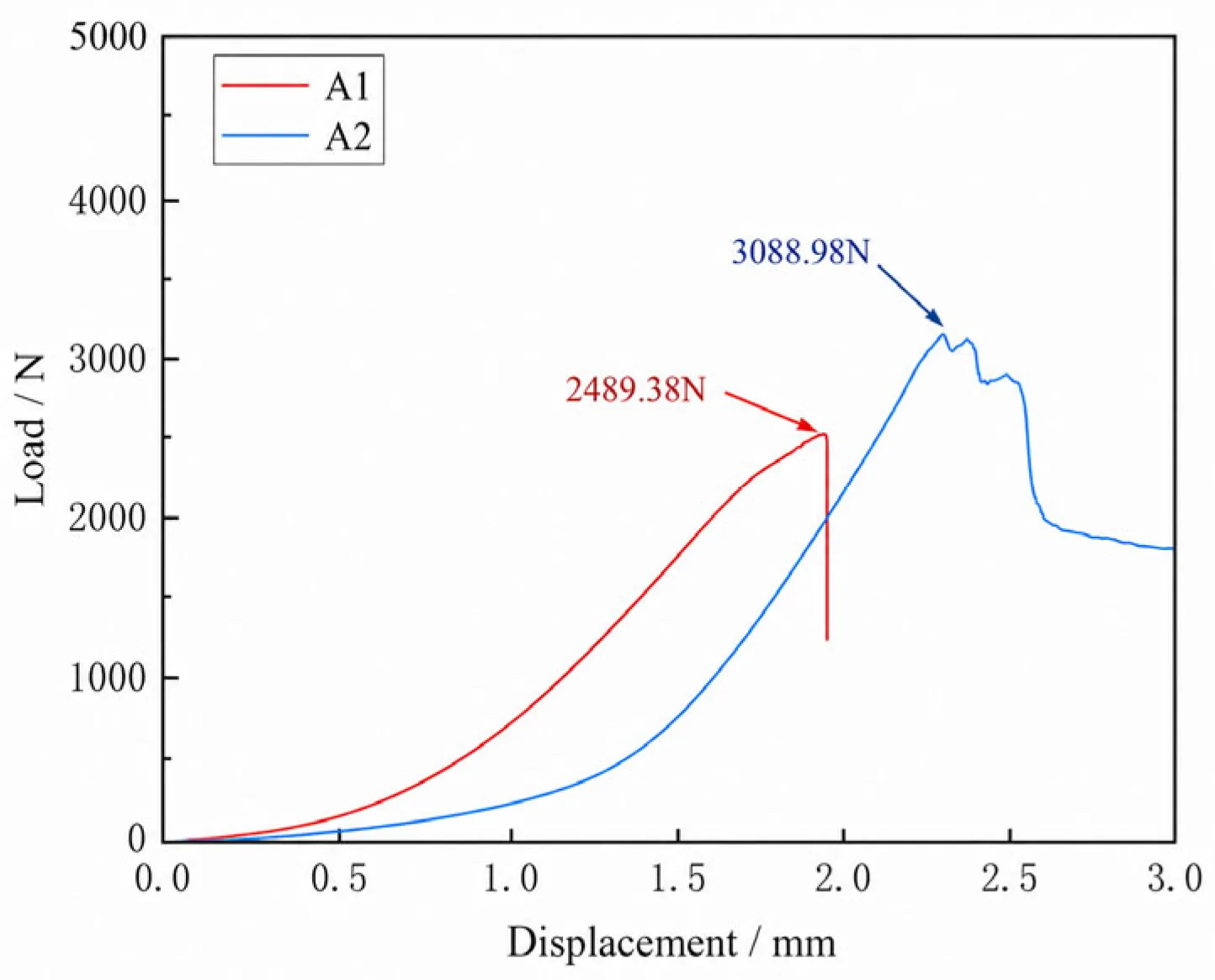
Load–Displacement Curves of Unreinforced Pipes.

**Figure 5:**
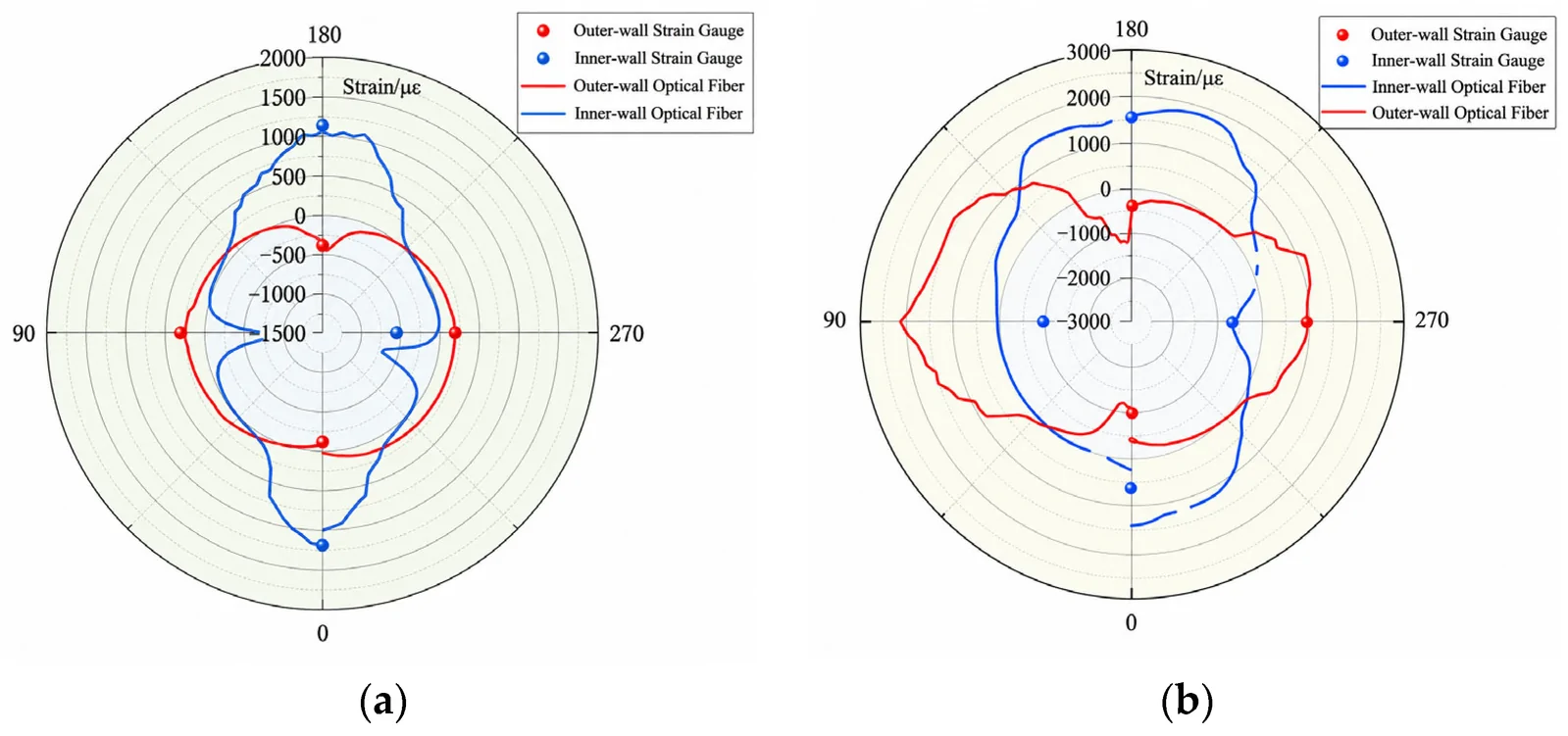
Hoop Strain of Group A Pipes: (**a**) Hoop strain of A1; (**b**) Hoop strain of A2.

**Figure 6:**
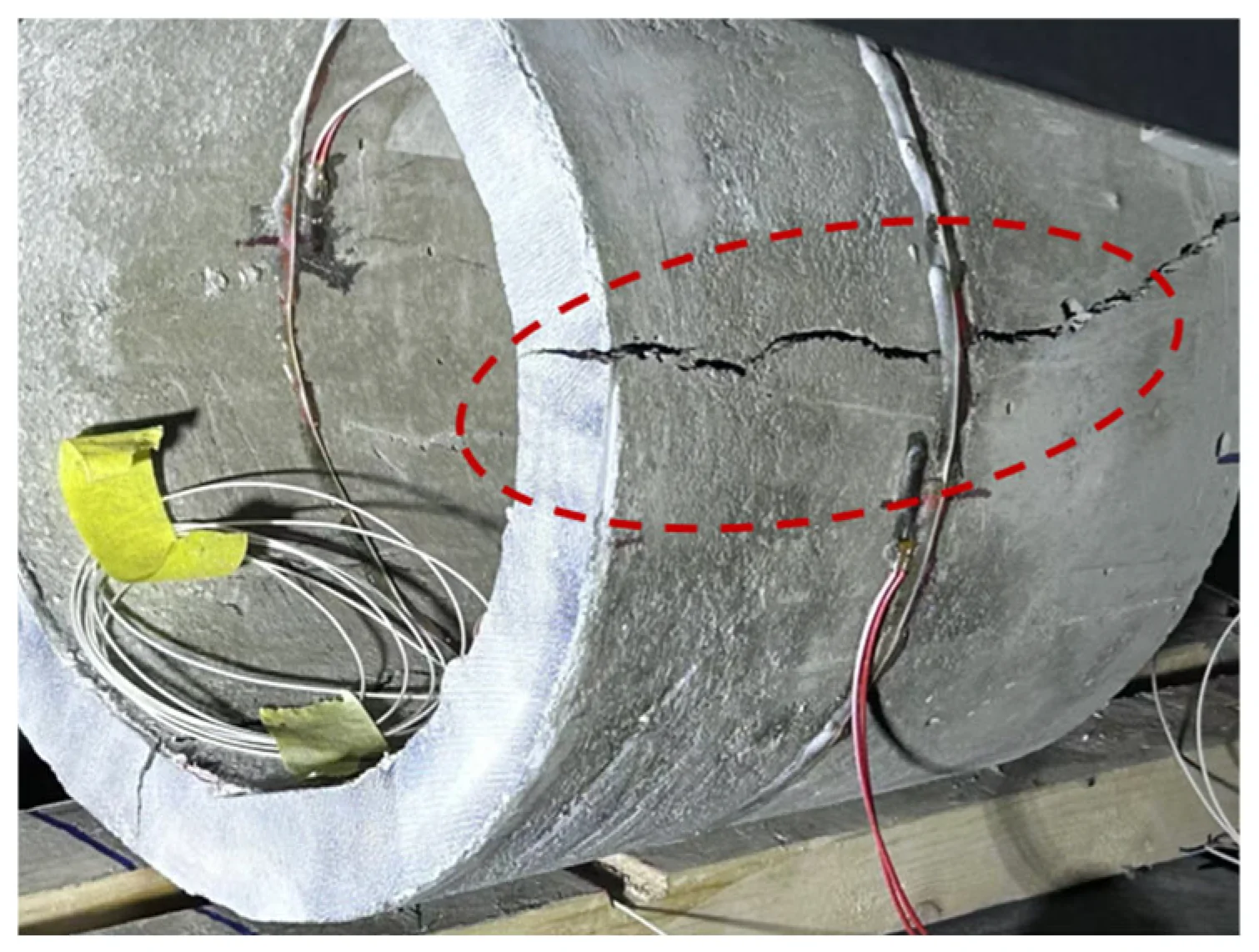
Through-Cracks in Unreinforced Pipes.

**Figure 7:**
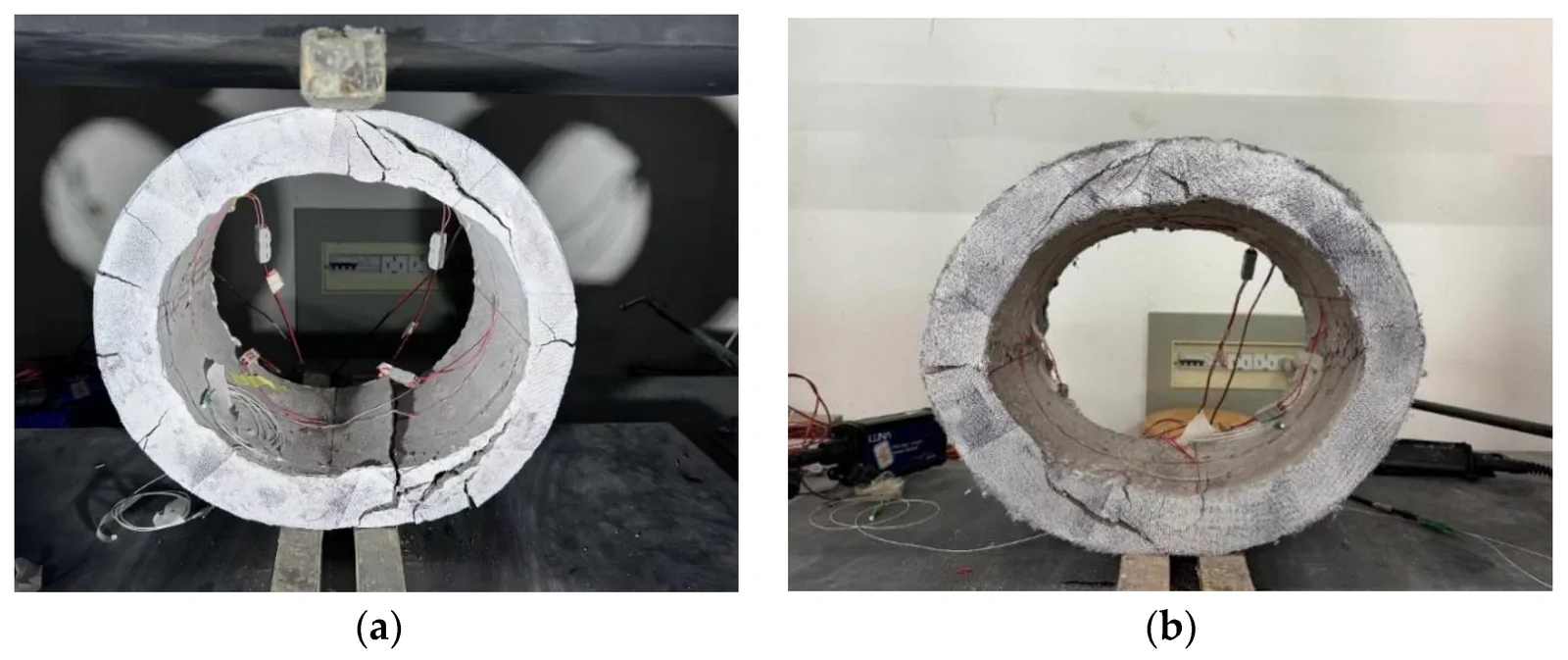
Final Failure Modes of Reinforced Pipes: (**a**) Experimental failure of B1 pipe; (**b**) Experimental failure of B2 pipe.

**Figure 8:**
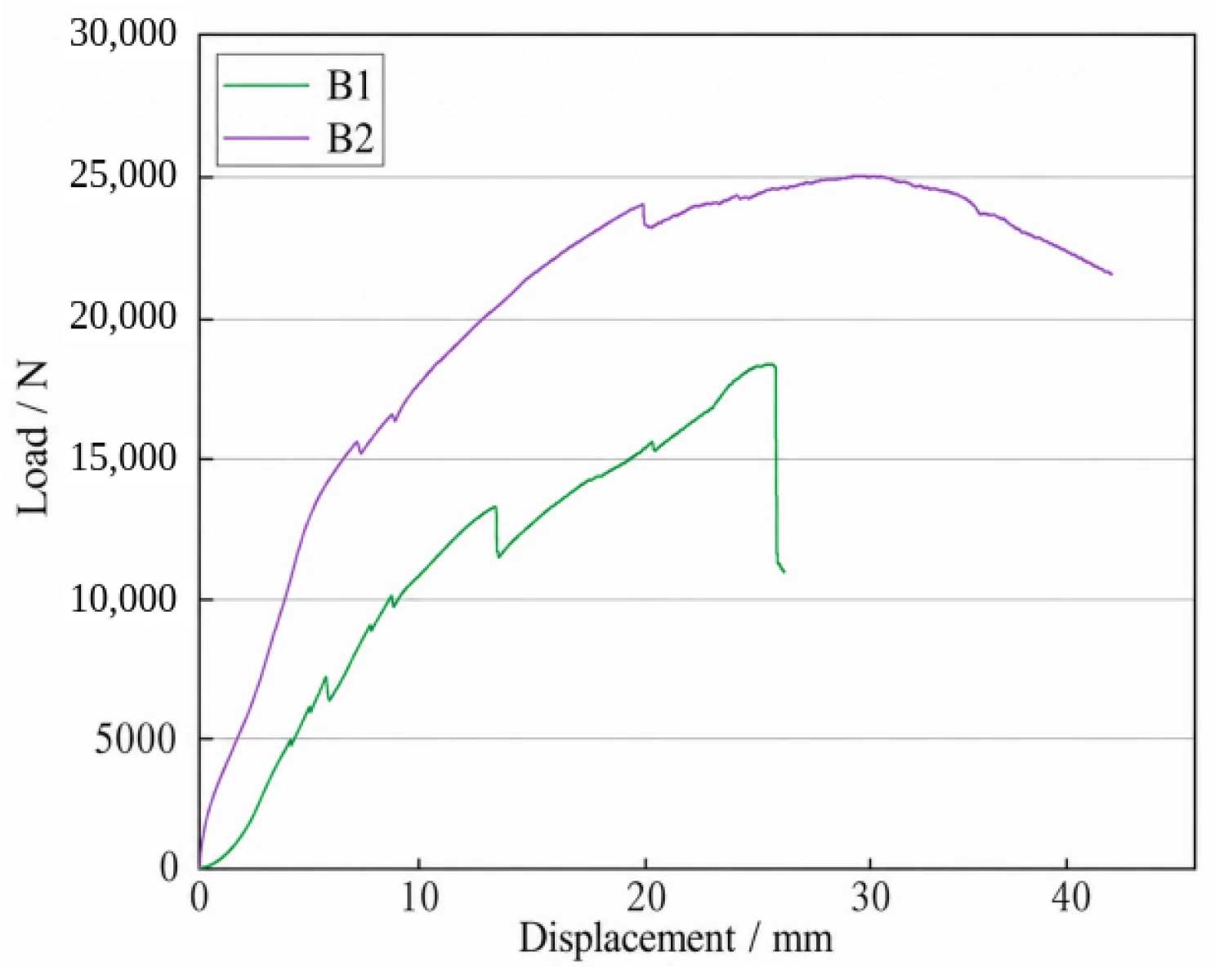
Load–Displacement Curves of Reinforced Pipes.

**Figure 9:**
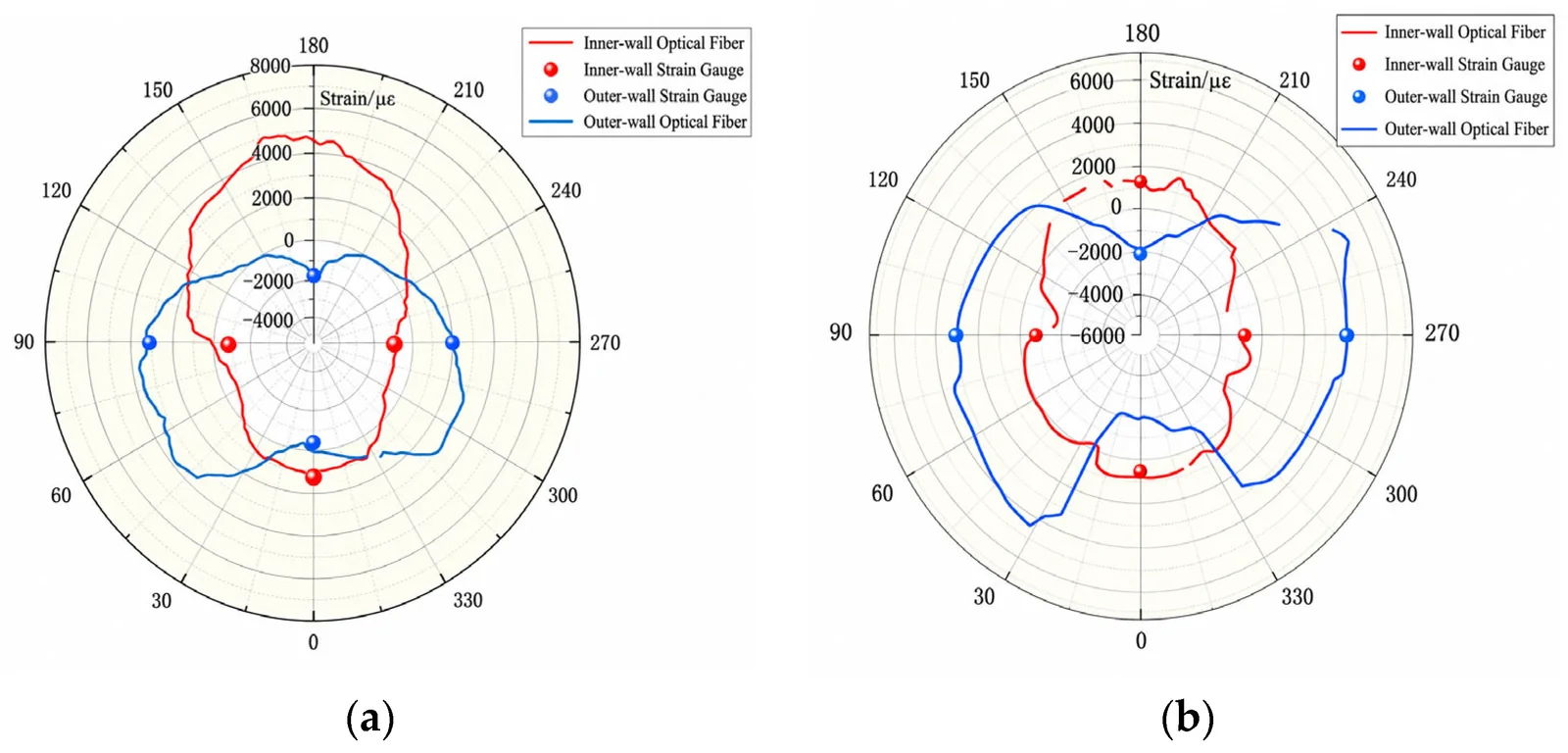
Hoop Strain of Group B Pipes: (**a**) Hoop strain of B1; (**b**) Hoop strain of B2.

**Figure 10:**
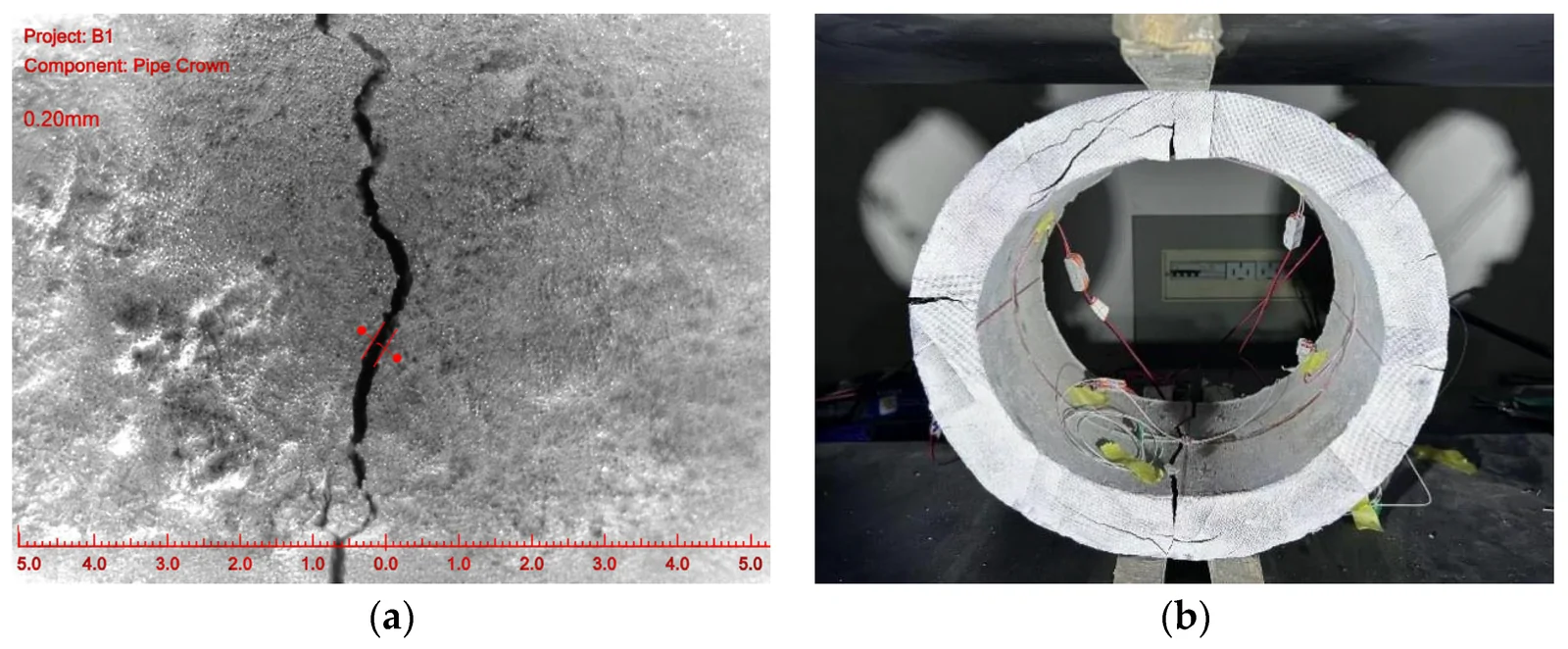
Crack Development Process of B1 Pipe: (**a**) Crack pattern when the crack width reached 0.2 mm; (**b**) front view of the crack failure.

**Figure 11:**
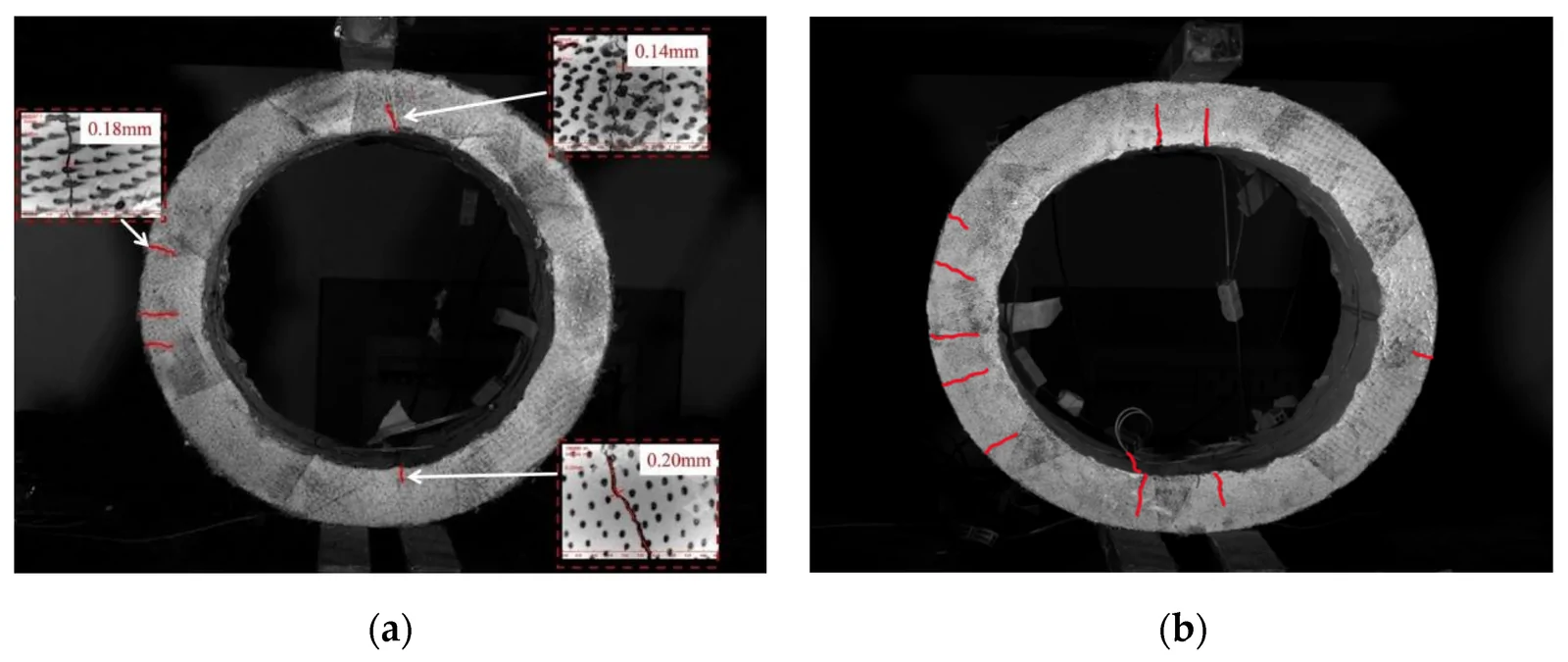
Crack Development Process of B2 Pipe: (**a**) Crack pattern when the crack width reached 0.2 mm; (**b**) front view of the crack failure.

**Table 1:** Parameters of Unreinforced Pipes.

Specimen No	Inner Diameter (mm)	Wall Thickness (mm)	Matrix Type
A1	200	25	EGC
A2	200	25	PVA-EGC

**Table 2:** Parameters of Reinforced Pipes.

Specimen No	Inner Diameter (mm)	Wall Thickness (mm)	Reinforcement Type	Longitudinal Bar Diameter (mm)	Stirrup Diameter (mm)	Reinforcement Cage Diameter (mm)	Matrix Type
B1	300	50	GFRP	6	6	350	EGC
B2	300	50	GFRP	6	6	350	PVA-EGC

**Table 3:** Material Properties of PVA-EGC.

Axial Compressive Strength (MPa)	Axial Tensile Strength (MPa)	Elastic Modulus (GPa)	Poisson’s Ratio	Elastic Limit Tensile Strain	Maximum Tensile Strain	Elastic Limit Compressive Strain	Maximum Compressive Strain
41.2	4.13	32.7	0.24	0.0001	0.025	0.004	0.006

**Table 4:** Material Properties of EGC.

Axial Compressive Strength (MPa)	Axial Tensile Strength (MPa)	Elastic Modulus (GPa)	Poisson’s Ratio	Elastic Limit Tensile Strain	Maximum Tensile Strain	Elastic limit Compressive Strain	Maximum Compressive Strain
34.4	3.07	30.8	0.22	0.0001	0.010	0.001	0.0035

## Data Availability

The original contributions presented in this study are included in the article. Further inquiries can be directed to the corresponding author.
